# A Versatile Route for Synthesis of Metal Nanoalloys by Discharges at the Interface of Two Immiscible Liquids

**DOI:** 10.3390/nano12203603

**Published:** 2022-10-14

**Authors:** Ahmad Hamdan, Luc Stafford

**Affiliations:** Département de Physique, Université de Montréal, 1375 Avenue Thérèse-Lavoie-Roux, Montreal, QC H2V 0B3, Canada

**Keywords:** nanosecond discharge, discharge in liquid, plasma-liquid interface, nanoalloys

## Abstract

Discharge in liquid is a promising technique to produce nanomaterials by electrode erosion. Although its feasibility was demonstrated in many conditions, the production of nanoalloys by in-liquid discharges remains a challenge. Here, we show that spark discharge in liquid cyclohexane that is in contact with conductive solution, made of a combination of Ni-nitrate and/or Fe-nitrate and/or Co-nitrate, is suitable to produce nanoalloys (<10 nm) of Ni-Fe, Ni-Co, Co-Fe, and Ni-Co-Fe. The nanoparticles are synthesized by the reduction of metal ions during discharge, and they are individually embedded in C-matrix; this latter originates from the decomposition of cyclohexane. The results open novel ways to produce a wide spectrum of nanoalloys; they are needed for many applications, such as in catalysis, plasmonic, and energy conversion.

## 1. Introduction

Reducing the dimension of a material to the nanoscale often reveals properties unattainable at the macroscopic level. Such discovery has emerged novel field of research: synthesis of nanomaterials. Be it 0D (e.g., nanoparticles), 1D (e.g., nanowires), or 2D (e.g., nanosheets), the properties of nanomaterials can be linked to enhanced chemical reactivity, remarkable electron and thermal transport, quantum behavior, etc. [1,2,3]. In addition to the dimension, chemical composition and spatial distribution of the elements play a crucial role on the functionalities of nano-objects [4,5,6]. In the case of nanoparticles, for example, core–shell and bi- or tri-metallic nanoalloys offer promising properties for many applications, including electronic, photonic, catalysis, and plasmonic [7,8].

Over the last two decades, several physical, chemical, or biological methods were developed to produce nanoalloys [9,10]. However, simple, versatile, and environmentally friendly techniques providing non-agglomerated and well-controlled nanostructures remain scarce. Gas-phase plasmas are often used in production of nanomaterials, including thermal as well as non-thermal processes [11,12]. For example, plasma torch is a well-known atmospheric pressure technique to produce various types of nanomaterials by processing solid, liquid, or suspension precursors [13,14,15]. Laser ablation is also used to generate nanomaterials through ablation of solid targets under different gas pressure conditions [16,17]. Very recently, plasma-liquid systems were proposed as novel method to efficiently produce nanomaterials [18,19]. The plasma can be either generated directly in liquid or in gas in contact with liquid [20]. The nanomaterials produced using the former type of discharges are strongly affected by both plasma-electrode and plasma-liquid interactions. In-liquid plasmas exhibit non-conventional properties of temperature (several thousands of Kelvin), pressure (several tens of bars), and density of charged species (10^17^–10^19^ cm^−3^) over very short time scales (rise and decay time less than 1 µs) [21,22,23,24]. Moreover, the flexibility of in-liquid plasmas allows discharge ignition in different liquids (e.g., water, hydrocarbons, cryogenics, etc.) and between electrodes of varying chemical nature (e.g., Al, Cu, Pt, Co, Ni, etc.) [25,26,27,28]. This has led to the production of a wide range of nanomaterials, including nanocomposites [27,29] and materials with novel crystallographic phases [30,31]. Although nanoalloys are searched for many applications, their synthesis is, however, not straightforward as it requires either multiple stage experiments [32] or the use of alloy electrodes [33,34].

Coupling a gas-phase plasma with a liquid that contain ions (supplied either by precursors or by metal electrode dissolution) was also proposed as efficient technique to produce nanomaterials through the reduction of the dissolved ions by the reactive species at the plasma-liquid interface. Using this approach, Velusamy et al. [35] synthesized CuO nanoparticles with a tailored energy-band diagram, Patel et al. [36] produced surfactant-free electrostatically stabilized gold nanoparticles, and Richmonds and Sankaran [37] synthesized Ag and Au nanoparticles. Recently, the feasibility of adding two kinds of ions (Eu and Ce) in solution was demonstrated to produce Eu doped Ce oxide nanoparticles by processing the mixture with the jet of an atmospheric pressure Ar plasma [38].

In a previous study, we introduced a novel plasma-liquid system, that combines the advantages of both in-liquid and in-contact with liquid systems. Indeed, discharges were sustained in liquid heptane that is in contact with a conductive solution (water + Ag-nitrate) [39]. This system successfully produced ultrasmall Ag nanoparticles embedded in a hydrocarbon network. The presence of carbon network is essential to prohibit the agglomeration between the nanoparticles and, therefore, to maintain their property as individual nanoparticles. Here, we examine the possibility to extent this technique for nanoalloys production. This is done by using solution with various kinds of metal salts (combination of Co-nitrate, Fe-nitrate, and Ni-nitrate), yielding non-agglomerated binary and ternary nanoalloys.

## 2. Materials and Methods

**Figure 1:**
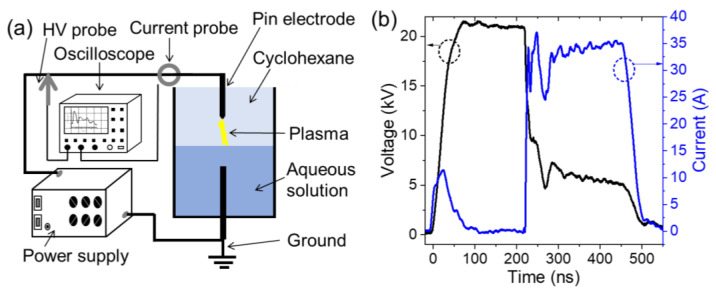
(**a**) Scheme of the experimental setup used to generate discharges in liquid cyclohexane that is in contact with a conductive solution. (**b**) Electrical characteristics (voltage and current) of a typical discharge in cyclohexane in contact with conductive solution (water + Co-nitrate + Ni-nitrate) generated at 22 kV voltage amplitude and 500 ns pulse width.

The experimental setup is schematically shown in Figure 1a. The discharge was ignited using a nanosecond positive pulsed generator (NSP 120-20-P-500-TG-H, Eagle Harbor Technologies, Seattle, WA, USA). The amplitude and width of the applied voltage were 22 kV and 500 ns, respectively. The discharge repetition rate was set to 10 Hz, and the duration of the experiment was 30 min. This low frequency has been chosen not to have correlation between two successive discharges through interaction between the bubble (induced by previous discharge) and the following discharge. Although this parameter has not been investigated here, we believe that it can be increased up to a few of kHz, which may significantly increase the yield of synthesis. The upper electrode, a carbon rod (99.99% pure; Goodfellow) that is mechanically polished to a curvature radius of ∼10  μm, was immersed in liquid cyclohexane, and the distance between its tip and the interface was kept at ∼1 mm. Meanwhile, the lower electrode, a carbon rod that is polished to a flat surface, was placed in the conductive solution at 3 mm below the interface. Note, the electrode erosion was insignificant. Current-voltage characteristics were recorded using a high-voltage probe (P6015A, Tektronix) for the applied voltage and a current coil (6585, Pearson) for the total current (discharge and displacement). Both probes were connected to an oscilloscope (DPO5420B, Tektronix) to record the corresponding voltage and current waveforms. Typical current-voltage waveforms for condition (i) (mixture of Co-nitrate and Ni-nitrate) are shown in Figure 1b. A drop in the voltage (from ~22 to ~5 kV) and a current peak (~35–40 A) can be seen; the initial current peak (~10 A in the period 0–50 ns) is due to the displacement current. Notably, rather identical characteristics were observed for all conductive solutions (not shown in Figure 1b). Usually, in-liquid spark discharge is characterized by a drop in the voltage to zero [40,41]. Here, the fact that the voltage drops to ~5 kV (and not to 0) indicates the discharge mode in not a conventional spark, but rather a spark-like. We believe that the presence of solution in electrical circuit adds a “new component” that should be considered in the analysis of the electrical characteristics. This can be conducted by, e.g., equivalent electrical circuit model, which is beyond the scope the study.

The synthesized nanostructures were characterized using a Transmission Electron Microscope (TEM, JEOL JEM-2100F) operated at 200 kV. For this purpose, the liquid samples collected after discharge treatment were sonicated for 5 min and then drop-casted on Cu TEM grids endowed with a lacey C-film (Electron Microscopy Science). The nanostructures were subsequently analyzed by bright field TEM imaging and Electron Dispersive Spectroscopy (EDS). After 30 min of discharge treatment at 10 Hz, both liquids change color, which indicates that they contain nanoparticles. In the previous study [39] where a solution of Ag-nitrate was used, Ag particles were synthesized in both liquids. Most particles collected from heptane were Ag nanoparticles (<10 nm) embedded in hydrocarbon network; meanwhile, the material collected from the silver nitrate solution was Ag nanoparticles (10–150 nm of diameter). Here, only the particles collected from cyclohexane side were characterized.

## 3. Results

**Figure 2:**
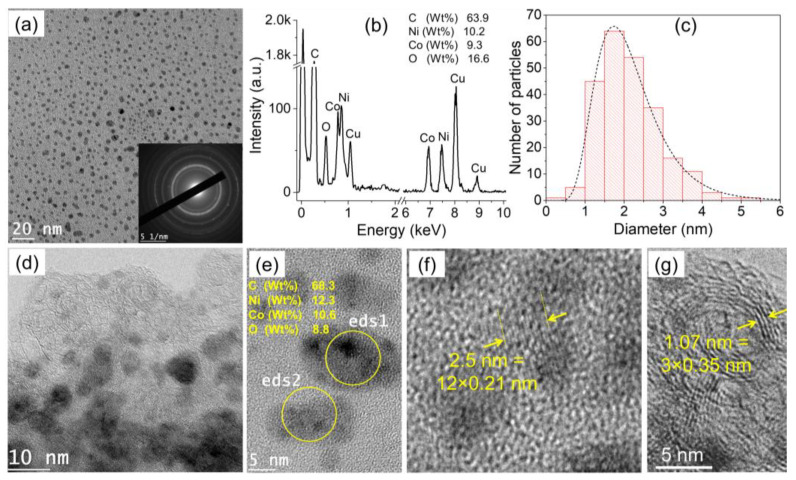
TEM characterization of the particles generated by discharges in cyclohexane in contact with water + Ni-nitrate + Co-nitrate collected from cyclohexane: (**a**) Low-resolution TEM image (inset: electron diffraction conducted on the imaged zone), (**b**) EDS spectrum acquired on the imaged zone in a), (**c**) size distribution of the synthesized nanoparticles, (**d**) intermediate-resolution TEM image, (**e**) high-resolution TEM image showing two typical nanoparticles as well as their composition deduced from EDS analysis, and (**f**,**g**) high-resolution TEM images showing the arranged atoms in the particles and in the matrix, respectively.

Figure 2 shows some morphological characteristics of the particles synthesized in condition (i), i.e., in a mixture of Co-nitrate and Ni-nitrate. Figure 2a is a low-resolution TEM image of the collected particles, and it clearly shows that they are not agglomerated. The inset is an electron diffraction pattern performed on the imaged zone; the rings indicate the crystalline nature of the nanoparticles. The imaged zone was also analyzed by EDS, and the obtained spectrum is shown in Figure 2b. The detected elements in the analyzed sample are C, O, Co, Ni, and Cu. Carbon is due to both, the lacey C-film on the TEM grid as well as to the matrix synthesized by the discharge due to its interaction with cyclohexane. Cu is also due to TEM grid. As for Co and Ni, they are due to the nanoparticles synthesized by the discharge. Finally, O is probably due to the oxidation of the nanoparticles. The oxidation may happen during synthesis (by the oxidative species during discharge such as OH and O) [39] or after being exposed to ambient air [42]. Figure 2c shows the particle size distribution deduced by measuring the diameter of many particles imaged by TEM. This distribution indicates that the majority of the particles have diameter between 1 and 5 nm.

Figure 2d is an intermediate TEM image showing that the produced nanoparticles are embedded in a film-like matrix. High-resolution TEM images are shown in Figure 2e–g. Local EDS analysis performed on two selected particles show very similar composition of 68.3 Wt% of C, 12.3 Wt% of Ni, 10.6 Wt% of Co, and 8.8 Wt% of O (Figure 2e). The high-resolution TEM image in Figure 2f highlights the arranged atoms in one particle; the measured interplanar distance (averaged on 12 layers) is ~0.21 nm, which could correspond to either Ni (111) or Co (111) interplanar distance (very close values 0.20 and 0.21 nm) [26]. Therefore, it is not possible to use such a measurement as criteria to differentiate Ni and Co distribution in a nanoparticle (this is also true when Fe is added). However, the local EDS analysis is more reliable, and it reveals the co-existence of Ni and Co in individual nanoparticles. Another high-resolution TEM image (Figure 2g) performed on the edge of the sample (as in Figure 2d) shows arranged structure of 0.35 nm interplanar distance, which corresponds to the graphitic carbon [43]. At this stage, one concludes that discharges in cyclohexane in-contact with a solution of Co-nitrate and Ni-nitrate produce individual (non-agglomerated) nanoalloy of Co-Ni embedded in carbon network.

**Figure 3:**
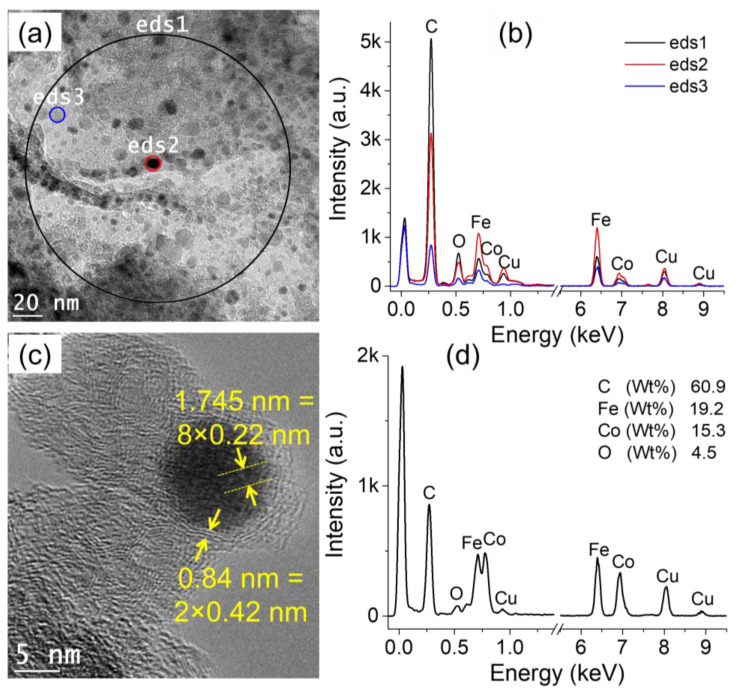
TEM characterization of the particles generated by discharges in cyclohexane in contact with water + Fe-nitrate + Co-nitrate collected from cyclohexane: (**a**) low-resolution TEM image, (**b**) EDS spectra acquired on the encircled zones in a), (**c**) high-resolution TEM image showing one nanoparticle and the matrix around it, and (**d**) EDS analysis performed on the imaged zone in (**c**).

Figure 3 shows the characteristics of the particles produced in condition (ii), i.e., in a mixture of Co-nitrate and Fe-nitrate. Figure 3a shows the low-resolution TEM image of the produced particles. EDS analyses were performed on a large region (eds1) as well as on selected nanoparticles (eds2 and eds3). The three spectra are shown in Figure 3b, and all of them show the presence of Fe and Co, in addition to C, O, and Cu. The particles size distribution was performed on the imaged particles (not shown here) and shows that the majority of the particles are <10 nm. Figure 3c is high resolution TEM image that shows one nanoparticle as well as a film-like structure in which the particle is embedded. The average interplanar distance measured in the nanoparticle is ~0.22 nm, while the interplanar distance of the film around the particle is around 0.42 nm. Local EDS analysis performed on the particle shows the presence of Fe and Co, in addition to the other species (Figure 3d). Here, also, one concludes that discharges in cyclohexane in-contact with a solution of Co-nitrate and Fe-nitrate produce individual nanoalloys of Co-Fe embedded in carbon matrix.

**Figure 4:**
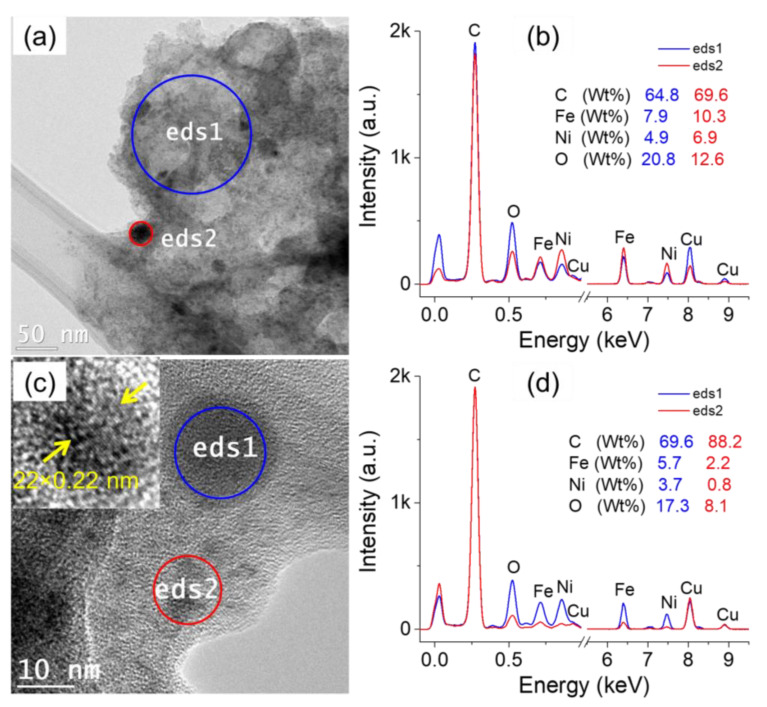
TEM characterization of the particles generated by discharges in cyclohexane in contact with water + Fe-nitrate + Ni-nitrate collected from cyclohexane: (**a**) low-resolution TEM image, (**b**) EDS spectra acquired on the encircled zones in a), (**c**) high-resolution TEM image showing individual nanoparticles and the matrix around it, and (**d**) EDS analysis performed on the encircled zones in (**c**).

Figure 4 summarizes the characteristics of the particles produced in condition (iii), i.e., in a mixture of Ni-nitrate and Fe-nitrate. The low-resolution TEM image (Figure 4a) shows that the majority of the particles are ultrasmall and are embedded in a film-like structure. Global and local EDS analysis were performed on the imaged zone, and the obtained spectra are shown in Figure 4b. Both spectra clearly show the presence of Fe and Ni, in addition to the other elements. A high resolution TEM image performed on the synthesized nanoparticles is depicted in Figure 4c. The measurement of the interplanar distance (average value) is ~0.22 nm (inset of Figure 4c). Local EDS analyses (Figure 4d) performed on a ~20 nm-diameter particle (eds1) as well as on ultrasmall particles (eds2) further indicate the presence of Fe and Ni in individual particles. As stated above, one concludes that discharges in cyclohexane in-contact with a solution of Ni-nitrate and Fe-nitrate produce isolated nanoalloys of Ni-Fe embedded in carbon network.

**Figure 5:**
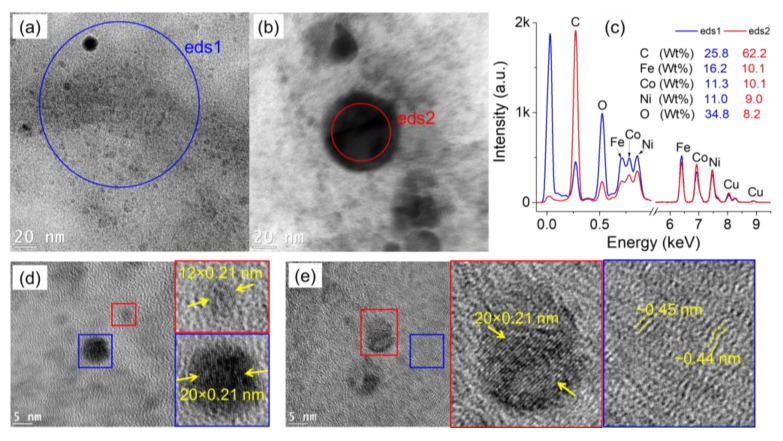
TEM characterization of the particles generated by discharges in cyclohexane in contact with water + Fe-nitrate + Ni-nitrate + Co-nitrate collected from cyclohexane: (**a**,**b**) low-resolution TEM images, (**c**) EDS spectra acquired on the encircled zones in (**a**,**b**), and (**d**,**e**) high-resolution TEM images showing individual nanoparticles and the matrix.

Finally, Figure 5 summarizes the observations performed on the particles synthesized in condition (iv), i.e., in a mixture of Ni-nitrate, Co-nitrate, and Fe-nitrate. TEM image (Figure 5a) shows that the majority of the particles are ultrasmall (<10 nm); it was also possible to find few large particles (50–100 nm), and a typical one is depicted in Figure 5b. EDS analysis performed on both particles’ population, the small as well as the large particles, shows the presence of Fe, Co, and Ni elements (Figure 5c), in addition to the other elements. Figure 5d,e show high-resolution TEM images of typical particles. The average interplanar distance measured on the particles is ~0.21 nm, and the measurement of the interplanar distance of the matrix is ~0.44–0.45 nm. These findings indicate, once again, that discharges in cyclohexane in-contact with a solution of Ni-nitrate, Co-nitrate, and Fe-nitrate produce nanoalloys of Ni-Fe-Co embedded in carbon matrix.

## 4. Discussion

The results presented above demonstrate that discharges in dielectric liquid (cyclohexane) that is in contact with conductive solution (water + metals nitrate) is a promising technology to efficiently produce non-agglomerated and well-controlled metal nanoalloys. All the four conditions tested here have led to various nanoalloys, namely Ni-Co, Ni-Fe, Fe-Co, and Ni-Fe-Co. The synthesis mechanisms are expected to be similar to those often highlighted in processes of plasma in-contact with solution [20]. Indeed, the reactive species in a typical gas phase plasma (e.g., electrons, radicals, etc.) reduce the ions in solution to form atoms that nucleate to form nanoparticles. Two major reactions are usually utilized to describe the growth [44]:

A^n+^ + ne → A^0^ (1)

A^n+^ + nH → A^0^ + nH^+^ (2)

where A^n+^ is an ion in solution (n = 3 for Fe-nitrate and 2 for Ni- and Co-nitrate), e is the electron, A^0^ is the reduced species, and H and H^+^ are the hydrogen atom and ion, respectively.

The simultaneous and homogeneous presence of multiple ions in solution induces a instantaneous reduction of the different ions, which then leads to their nucleation as nanoparticles. On the other hand, the decomposition of cyclohexane by plasma produces many carbonaceous species (e.g., C_2_, C_x_H_y_, etc.) that contribute to the formation of the carbon matrix. Because of the variety of carbon structures observed here, it is not straightforward to advance its mechanisms of formation. However, in some specific cases (e.g., Figure 2g and Figure 3c), it is possible to identify carbon structures grown around the particle, which could be related to catalysis effect. Indeed, as it is well known in chemical vapor deposition (CVD) techniques, the growth of carbon nanostructures (e.g., nanotubes) requires the presence of three ingredients: carbon precursor, high temperature, and catalysis [45,46]. Over the range of experimental conditions investigated, all the three ingredients are present, such that it can be proposed that the specific carbon nanostructures (Figure 2g and Figure 3c) are related to a CVD-like growth. Furthermore, it is worthy to note that the production of the matrix, simultaneously with the nanoparticles, reduces the particle-particle interactions, which prohibits their agglomeration. Such characteristic is extensively searched for, especially in the field of deposition of multifunctional nanocomposite thin films [47,48]. Note that the production of nanoalloys using the present process may be used in other processes to obtain devices with specific properties (e.g., catalytic properties). In the cases when the presence of carbon matrix is undesired, we believe that it can be removed by an appropriate method before or during processing. Finally, this study is considered as proof-of-concept to produce metal nanoalloys by a novel discharge-based technique. The production yield may be enhanced by increasing, e.g., the discharge frequency. Furthermore, instead of conducting discharges in stationary liquids, it is also feasible to conduct discharges in microfluidic devices. Although this has not been tested so far, we believe that it can be one of the methods to produce nanoalloys at large-scale.

## 5. Conclusions

In summary, compared with conventional synthesis techniques, in-liquid discharges offer some advantages and deserve further development. First, in-liquid discharges produce transient plasmas with high temperature (several thousands of Kelvin), high pressure (several tens of bar), and high density of reactive species (e.g., electron density of ~10^17^–10^19^ cm^−3^), which significantly increases the yield of synthesis. Second, the upper liquid (here cyclohexane) may be used as additional precursor, and it contributes to the final product. Therefore, its composition could be adjusted to fit a targeted material. We are convinced that such discharge conditions open the way and can be used as building blocks to synthesize ‘novel’ nanomaterial, probably with novel properties. In terms of perspective, additional systematic work should be conducted with the aim to address the influence of the relative concentration of different ions in water on the final product; this parameter may be used to finely control the composition of the nanoalloys.

## Data Availability

The data that supports the findings of this study are available within the article.
